# *SAP97* regulates behavior and expression of schizophrenia risk enriched gene sets in mouse hippocampus

**DOI:** 10.1371/journal.pone.0200477

**Published:** 2018-07-11

**Authors:** Preetika Gupta, Ogul E. Uner, Soumyashant Nayak, Gregory R. Grant, Robert G. Kalb

**Affiliations:** 1 Neuroscience Graduate Group, Department of Neuroscience, University of Pennsylvania, Philadelphia, Pennsylvania, United States of America; 2 School of Arts and Sciences, Department of Biology, University of Pennsylvania, Philadelphia, Pennsylvania, United States of America; 3 Institute for Translational Medicine and Therapeutics, University of Pennsylvania, Philadelphia, Pennsylvania, United States of America; 4 Department of Genetics, University of Pennsylvania, Philadelphia, Pennsylvania, United States of America; 5 Feinberg School of Medicine, Department of Neurology, Northwestern University, Chicago, Illinois, United States of America; University of Queensland, AUSTRALIA

## Abstract

Synapse associated protein of 97KDa (*SAP97*) belongs to a family of scaffolding proteins, the membrane-associated guanylate kinases (MAGUKs), that are highly enriched in the postsynaptic density of synapses and play an important role in organizing protein complexes necessary for synaptic development and plasticity. The Dlg-MAGUK family of proteins are structurally very similar, and an effort has been made to parse apart the unique function of each Dlg-MAGUK protein by characterization of knockout mice. Knockout mice have been generated and characterized for *PSD-95*, *PSD-93*, and *SAP102*, however *SAP97* knockout mice have been impossible to study because the *SAP97* null mice die soon after birth due to a craniofacial defect. We studied the transcriptomic and behavioral consequences of a brain-specific conditional knockout of *SAP97* (SAP97-cKO). RNA sequencing from hippocampi from control and SAP97-cKO male animals identified 67 *SAP97* regulated transcripts. As large-scale genetic studies have implicated MAGUKs in neuropsychiatric disorders such as intellectual disability, autism spectrum disorders, and schizophrenia (SCZ), we analyzed our differentially expressed gene (DEG) set for enrichment of disease risk-associated genes, and found our DEG set to be specifically enriched for SCZ-related genes. Subjecting SAP97-cKO mice to a battery of behavioral tests revealed a subtle male-specific cognitive deficit and female-specific motor deficit, while other behaviors were largely unaffected. These data suggest that loss of *SAP97* may have a modest contribution to organismal behavior. The SAP97-cKO mouse serves as a stepping stone for understanding the unique role of *SAP97* in biology.

## Introduction

Intellectual disabilities and neuropsychiatric behavioral disorders affect about 17.9% of individuals over their lifetime and interfere with the ability of people to experience a fulfilling and productive life (nimh.nih.gov). Some of these disorders are clearly developmental. For example, autism spectrum disorders (ASD) are characterized by impairments in social interaction and communication, and by restricted, repetitive behaviors and about 1% of children show signs and symptoms that lead to the diagnosis of ASD [1,2]. Schizophrenia (SCZ) is another mental disorder that is characterized by disordered thought processes and disturbed emotional responsiveness [3]. The symptoms of SCZ usually appear during young adulthood, with an overall prevalence of about 0.7% [3,4]. Technological advances have brought unprecedented insights into the genetic architecture of these and many other neuropsychiatric disorders [4–7].

Exome-sequencing technology has allowed us to systematically scan genes for *de novo* mutations at the single-base resolution, potentially offering insights into risk-determining genes [4,8]. Whole-exome sequencing results from patients with ASD or SCZ reveal significantly enriched copy number variant (CNV) mutations in the synaptic gene set [4]. Among the most prevalent synaptic genes that have been uncovered in large-scale genomic studies have been alterations in the *neurexins/neurolignins* along with the *ProSAP/Shank* family. Various genetically manipulated mice of these gene families recapitulate some of the behavioral features of ASD, SCZ, and intellectual disability [9–12]. However, none of these models completely phenocopy disease in humans, consistent with the polygenic origin of these disorders.

Another important group of synaptic genes that has been implicated to be involved in ASD or SCZ is the Discs-large (Dlg) family of membrane associated guanylate kinases (MAGUKs) [7,13–16]. The Dlg family is the most comprehensively studied family of MAGUKs, and is comprised of *PSD-95*, *PSD-93*, *SAP102*, and *SAP97*. They share a common domain structure comprised of three PDZ domains, along with an SH3 and GUK domain. The Dlg-MAGUK family directly binds to many proteins in the postsynaptic density (i.e. glutamate receptor subunits, TARPS, and neurexin/neuroligin clusters), and regulates synaptic nanoscale structure and synaptic transmission [17–19] . Mice with a targeted deletion of *PSD-95*, *PSD-93*, and *SAP102* show a range of phenotypes also displayed by individuals with psychiatric disorders [15,16,20].

A variety of evidence implicates *SAP97* in the etiology of ASD and SCZ: 1) single nucleotide polymorphisms in *SAP97* have been linked to an increased risk of schizophrenia in males [21], 2) a more recent genetic association analysis detected an association between schizophrenia and single nucleotide polymorphisms located within a newly identified primate-specific exon of *SAP97* [22], 3) individuals with 3q29 microdeletions spanning the *SAP97* locus display autism and intellectual disability [23], 4) a meta-analysis demonstrated the 3q29 deletion confers a 40-fold increased risk for SCZ [24], and 5) a study of expression levels of multiple postsynaptic density proteins found a specific decrement in the level of *SAP97* in post mortem frontal lobe from schizophrenic patients [25]. *SAP97* is also the only member of the Dlg-MAGUK family that directly binds to the extreme C-terminus of the *GluA1* AMPA receptor (AMPAR), a subunit that promotes dendritic growth and patterned synaptic innervation [26,27]. Thus, it is plausible that defects in these *SAP97*-dependent mechanisms contribute to a ASD and SCZ phenotype. While these findings advocate for the participation of *SAP97* in the etiology of neuropsychiatric disorders, the issue has been difficult to study because SAP97 knockout mice die a few days after birth from a craniofacial defect [28].

In this study, we chose to pursue a broad behavioral and transcriptomic characterization of *SAP97* deficient mice to gain an overall understanding of the role of *SAP97* in brain function and organismal level. We generated mice that have a conditional knockout of *SAP97* targeted to neurons using the Cre-loxP system. We then characterized these mice at the behavioral and biochemical level. Overall, our results suggest that loss of *SAP97* results in mild sex-specific behavioral abnormalities as well as regulates transcripts of SCZ risk-related genes. This study provides us with the framework to conduct subsequent studies that can address whether *SAP97* directly contributes to the pathophysiology of SCZ.

## Materials and methods

### Animals

All animal procedures were approved by the Children’s Hospital of Philadelphia Institutional Animal Care and Use Committee. Mice were housed (2–5 animals per cage) with a 12/12 hr light/dark cycle and with ad libitum access to food and water. Approximately 100 total animals were used for this study. The Cre-loxP system was used to generate a SAP97 conditional knockout (cKO) mouse. SAP97fl/- mice were generated as previously described. Nestin-cre+/- mice on a C57Bl/6 background were purchased from Jackson Labs (stock number 003771). Nestin-cre+/-; SAP97fl/- mice were generated by crossing male Nestin-cre+/- with female SAP97fl/- mice. Nestin-cre+/-; SAP97fl/- male mice were then crossed with female SAP97fl/- mice to generate Nestin-cre+/-; SAP97fl/fl (SAP97-cKO) and littermate control animals. Littermate control animals (genotype: Nes-cre+/-, SAP97fl/fl, and wild-type) were averaged and compared to cKO animals. Genomic DNA was extracted from tail snips (obtained at 10 days of age) using the Phenolcholoroform acetate method to confirm genotypes. The primers used for genotyping were as follows: *SAP97* flox fwd- AGAGTATGCTCTATGTGATGTTGTGTG rev-TAAGAAGGATCAACTGGCAAAGGTG; *CRE* fwd- ACCTGATGGACATGTTCAGG rev-CGAGTTGATAGCTGGCTGG

### Behavioral experiments

#### Open field

Assessment of general exploratory behavior was evaluated using the open field paradigm. Mice were placed in a white, opaque plexiglass box (40cm x 40cm) and were given 15 minutes to explore the apparatus. Exploratory locomotor activity (total distance traveled and average speed) was scored using the Any-MAZE tracking software (San Diego Instruments, San Diego, CA).

#### Elevated plus maze

Assessment of anxiety-like behaviors was evaluated using an elevated plus maze (Coulbourn Instruments, Whitehall, PA). The mouse was initially placed in the center “free zone”, and was allowed to freely explore the apparatus for the 5-minute trial time. Time in the open arms versus the closed arms, as well as number of entries to these arms, was measured using the Any-MAZE tracking software.

#### Accelerating rotarod

Assessment of motor learning and motor coordination was evaluated using the accelerating rotarod (Ugo Basile, Varese, Italy). The starting acceleration was 4 rpm, and accelerated to 40 rpm over a 5-minute trial time. Mice underwent 3 trials per day for 4 consecutive days, for a total of 12 trials. Latency to fall from the rod was manually measured and compared across the 4 days.

#### Novel Object Recognition (NOR)

Assessment of cognition was evaluated using the NOR paradigm. The testing apparatus was a white, opaque plexiglass box (40cm x 40cm). On day 1, mice were habituated to the testing apparatus for 15 minutes. On day 2, the mice were reintroduced to the testing apparatus and allowed to explore two identical objects equally spaced from the walls of the apparatus (objects A and A’) for 5 minutes and the animal was then removed. Any-MAZE tracking software was used to measure the time spent investigating each object, and a preference index (PI) was calculated by dividing time spent investigating A’ by time spent investigating A (A’/A). One hour later after identical object exploration, the mouse was placed back in the testing apparatus where one of the identical objects had been replaced with a novel object that differed in shape, color, and texture (object B). Again, the mouse was given 5 minutes to explore the two objects, and preference index for the novel object was calculated by dividing time spent investigating B by time spent investigating A (B/A). Significant preference for the novel object was assessed by comparing the PI from the training phase to the PI from the testing phase.

#### Three chambered social choice

Assessment of sociability was evaluated using the standard three-chambered social choice paradigm. A white, opaque plexiglass rectangular box was used, with three partitions (each 20cm x 40cm). The mouse was first given 5 minutes to habituate to the empty apparatus. After habituation, into the left and right compartments was placed either with an inanimate object (nonsocial zone) or an age and sex-matched C57Bl/6 mouse (social zone). The object and mouse were placed under clear, plexiglass cylinders with perforations to allow odor detection. During the testing phase, the test mouse was allowed five minutes to explore either zone. Time in each zone was measured using the Any-MAZE tracking software. Zone preference index was calculated by dividing specific zone time (either social or nonsocial) by total zone exploration time.

### Biochemistry

Mice were anesthetized with a pentobarbital solution and decapitated. The brain was removed, and each hemisphere of the cerebellum, cerebral cortex, and hippocampus was dissected. One hemisphere was rapidly transferred to a mortar and pestle prechilled on dry ice, and ground into a fine powder to be processed for RNA extraction by the RNeasy mini kit (Qiagen, Catalogue #74134) according to the manufacturer’s instructions. Once RNA extraction was complete, conversion to cDNA was done using the iScript Supermix (Bio-Rad, Catalogue #1708841, Hercules, CA). The other hemisphere was transferred to a dounce prechilled on ice, and lysed in 1% Triton-X lysis buffer (150mM NaCl, 50mM Tris-HCl pH 8.0, 1mM EGTA, 5mM EDTA) with protease and phosphatase inhibitors for generation of protein lysates.

### Antibodies

The following antibodies were used in this study as follows: immunoblotting of *SAP97* (Thermo Fisher Scientific, catalogue # PA1-741); immunoblotting of *PSD95* (NeuroMab, catalogue # 75–348); immunoblotting of *PSD93* (NeuroMab, catalogue # 75–284); immunoblotting of *SAP102* (NeuroMab, catalogue # 75–058); immunoblotting *beta-actin* (Cell Signaling Technology, catalogue # 3700 (mouse), or Sigma-Aldrich, catalogue # A2066 (rabbit)). Secondary antibodies for immunoblots (IRDye) were purchased from Li-COR (Catalogue # 925–32210, and Catalogue # 925–68021).

### Western blot

Western blot was performed according to standard procedures [29–31].

### Quantitative PCR

Quantitative real-time PCR (qPCR) was carried out as previously described using the delta delta Ct method to calculate relative gene expression levels [32]. Ribosomal S17 and S18 (RS17, RS18) were used as reference genes. Each reaction consisted of cDNA, primers, and Power SYBR Green PCR Master Mix (Applied Biosystems, Catalogue # 4367659, Waltham, MA) with a total 25uL reaction volume. Melting curve analysis of the target sequences showed that all primers used in this study generated amplification of a single peak, without primer-dimer artifacts. Primer and cDNA concentrations were optimized prior to use in qPCR experiments. Each qPCR experiment consisted of 4–6 biological replicates, as well as three technical replicates per sample. The primers used for qPCR included: *GLUA1* fwd- CCCTGAGAGGTCCCGTAAAC rev- GCTCAGAGCACTGGTCTTGT; *GLUA3* fwd- CCATGCTCTTGTCAGCTTCG rev- AGTCCACCTATGCTGATGGT; *GLUA4* fwd- TGAATGAACAAGGCCTCTTGGA rev- AGGCACTCGTCTTGTCCTTG; *NRCAM* fwd- AAGACCCGCTGGACTTTGAA rev- GGCTTGCCATTGCCTTCTTA; *HUWE1* fwd- GTTGGGATTTCCCACCAGGA rev- CAGTCTGCAGGAGCTTCAGT; *PTEN* fwd- CCTGCAGAAAGACTTGAAGGTG rev- CTGTGCAACTCTGCAGTTAAA; *ADAM10* fwd- GGCTGGGAGGTCAGTATGGA rev- CTCGTGTGAGACTGCTCGTT; *WAS* fwd- TCAGCTGAACAAGACCCCTG rev- CATGCATCAGGGCACCTACT; *ERBB4* fwd- ACCCAGGGGTGTAACGGT rev- TGGTAAAGTGGAATGGCCCG; *SEMA4C* fwd- GGTGGCCGGAGTCAAACG rev- TTCAGTCCAGCAGCCCTCTTT; *KCNA3* fwd- TCCGAAAAGCCCGGAGTAAC rev- CTGTGGAGTTGCCCGTTTTG; *KCNA4* fwd- CACTTGCTGGGAATGGTGAAGT rev- GAGAAGGTGGTAGACGCAGT; *KCNA5* fwd- TAGGACACTGGCTGACCCAT rev- ACGCACAAGCAGCTCAAAAG; *GNG13* fwd- TTGCTGTCTCCTCCAAAACCTC rev- TCCCTCTTGAAGGCCAGTTG; *FZD7* fwd- AGAACCTCGGCTACAACGTG rev- ACCGAACAAAGGAAGAACTGC; *DLGAP4* fwd- TTTGCTTCTCTGCCCGATCC rev- TGATGAACATTGCTTCAAGAGC; *CTNNA1* fwd- CAGTTCGCTGCAGAAATGAC rev- ACCTGTGTAACAAGAGGCTCC; *CALM3* fwd- GAGTAACCTCGATCCCCGAG rev- GAAGGCTTCCTTGAACTCTGC; *KCNC1* fwd- CTACGCGCGGTATGTGGC rev- TCGGTCTTGTTCACGATGGG; *S18* fwd- CAGCTCCAAGCGTTCCTGG rev-GGCCTTCAATTACAGTCGTCTTC; *S17* fwd- GATTCAGAGAGGGCCTGTGAG rev-CTGAGACCTCAGGAACGTAGT

### RNA sequencing

RNA was isolated from four control and four SAP97-cKO male hippocampi, quality evaluated by Bioanalyzer 2100 (Agilent Technologies, Santa Clara, CA), and sequenced with an Illumina HiSeq 4000 High-Throughput Sequencing System. The RNA-seq reads were aligned to the mouse genome mm10.GRCm38.p5 using STAR version 2.5.3a [33]. Next, normalization and quantification were performed with the PORT version 0.8.2a-beta pipeline (http://github.com/itmat/Normalization) which first removes reads that map to ribosomal RNA sequences or mitochondrial DNA and then uses a read re-sampling strategy for normalization to account for batch effects and differences in sequencing depth among the samples. After the normalization procedure, the gene level quantification was done by PORT with respect to the Ensemblv90 annotation. The normalized count of reads mapping to exon 10 of *SAP97* showed almost 30-fold reduction from an average of 242 in the control samples to an average of 8 in the SAP97-cKO samples affirming the efficacy of the knockdown procedure. The differential expression analysis was performed using the R Bioconductor package limma-voom [34,35]. The top 566 genes with FDR < 0.5 and fold-change of greater than 1.6 were used for general pathway enrichment analyses, which were performed using Ingenuity IPA.

### Analysis of overlap between differentially expressed genes and risk-associated disease genes

The significance of overlap between the set of differentially expressed genes (DEGs) and risk-associated ASD, SCZ, and other neuronal disorder genes was analyzed using non-parametric analysis. The ASD gene list was chosen from research by Silvia De Rubeis et al., while the SCZ and ataxia gene lists were chosen from online resources (szdb.org, genedx.com). Details of the lists chosen and overlap analysis are discussed in results section. The mean and variance of the corresponding hypergeometric distribution were calculated. The *p* value of the significance of the overlap was estimated using the hypergeometric probability test.

### Statistics

Data were analyzed using Prism (GraphPad Software, La Jolla, CA). Significant differences within groups were determined using either Student’s t-test, one-way ANOVA followed by Tukey’s test for multiple comparisons, or repeated-measures two-way ANOVA followed by Tukey’s test for multiple comparisons. For all tests except for RNAseq, the significance threshold was set to p<0.05. The significance threshold for DEGs in the RNAseq experiment was set to FDR <0.25.

## Results

### Targeted deletion of SAP97 to neurons

**Fig 1:**
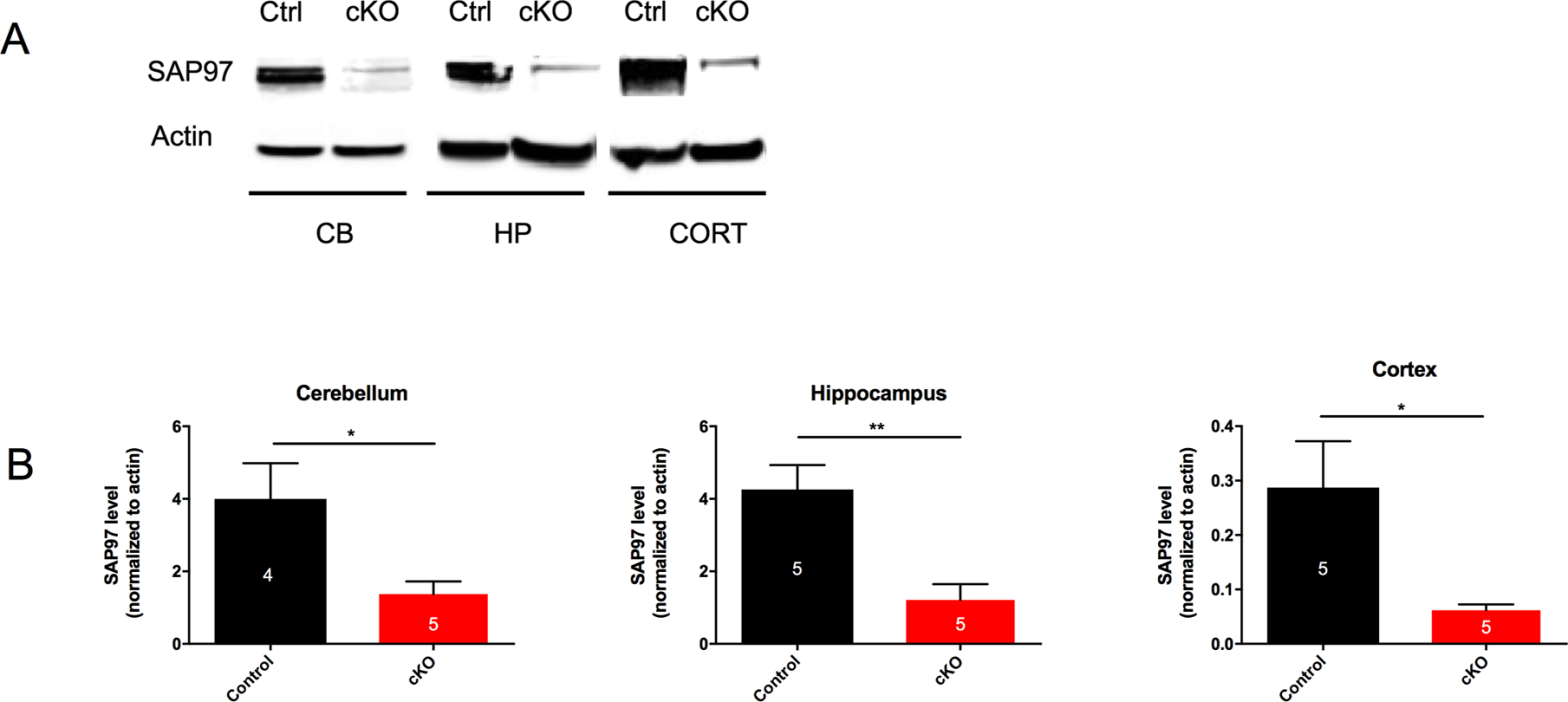
SAP97 protein is sufficiently knocked down in SAP97-cKO animals. (A) Western blots showing reduced *SAP97* band intensity in cerebellum, hippocampus, and cortex. (B) Quantification of western blot analysis. *P < .05, **P < .01 (two-tailed Student’s *t* test). Data are presented as mean ± SEM.

Global *SAP97* knockout mice have been generated, but die soon after birth due to a craniofacial defect. In order to study the effect of loss of *SAP97* on neuronal development and behavior, we conditionally knocked out *SAP97* by crossing Nestin-cre mice with *SAP97* floxed mice (see Methods). SAP97-cKO mice were born at Mendelian ratios and were grossly normal. At two months of age, we harvested tissue from the cerebellum, hippocampus, and cortex from control and SAP97-cKO animals of both sexes and prepared protein lysates for western blot analysis. In all three brain regions, the abundance of *SAP97* was significantly reduced (Cerebellum: Ctrl 3.998 ± 0.9833, n = 4; SAP97-cKO 1.373 ± 0.348, n = 5, p = 0.0279; Hippocampus: Ctrl 4.252 ± 0.6751, n = 5; SAP97-cKO 1.215 ± 0.4314, n = 5, p = 0.0053; Cortex: Ctrl 0.287 ± 0.08557, n = 5; SAP97-cKO 0.06179 ± 0.01072, n = 5, p = 0.0311), indicating that we successfully generated cKO animals (Fig 1).

### No apparent compensation by other Dlg-MAGUK family members

**Fig 2:**
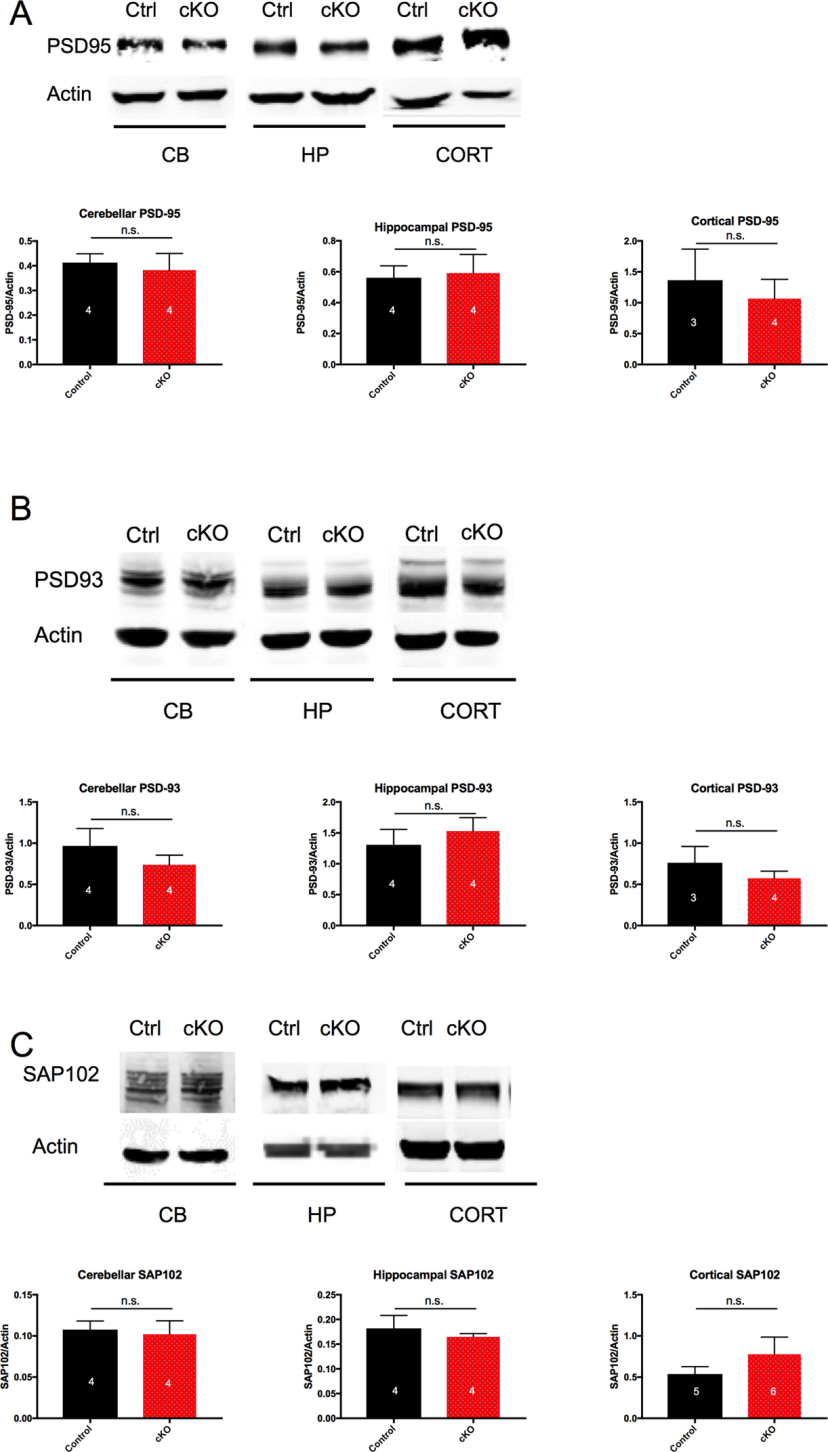
No compensation by Dlg-MAGUK family abundance in SAP97-cKO animals. (A) Western blots and quantification showing no significant change in abundance of *PSD-95* in cerebellum, hippocampus, and cortex. (B) Western blots and quantification showing no significant change in abundance of *PSD-93* in cerebellum, hippocampus, and cortex. (C) Western blots and quantification showing no significant change in abundance of *SAP102* in cerebellum, hippocampus, and cortex. n.s., no significance (two-tailed Student’s *t* test). Data are presented as mean ± SEM.

Previous work shows that the Dlg-MAGUKs (*PSD-95*, *PSD-93*, *SAP102*, and *SAP97*) can have redundant functions in electrophysiological assays. In order to examine whether loss of *SAP97* led to compensatory changes in the abundance of the other Dlg-MAGUK family members, we measured total protein levels in the cerebellum, hippocampus, and cortex. There was no significant difference in the abundance of any other Dlg-MAGUK at the protein level in control versus SAP97-cKO male animals (Fig 2). These data suggest that if members of the Dlg-MAGUK family compensate for the lack of *SAP97*, they do so without a change in overall abundance.

### No changes in gene expression level of known SAP97 binding partners or interactors

**Fig 3:**
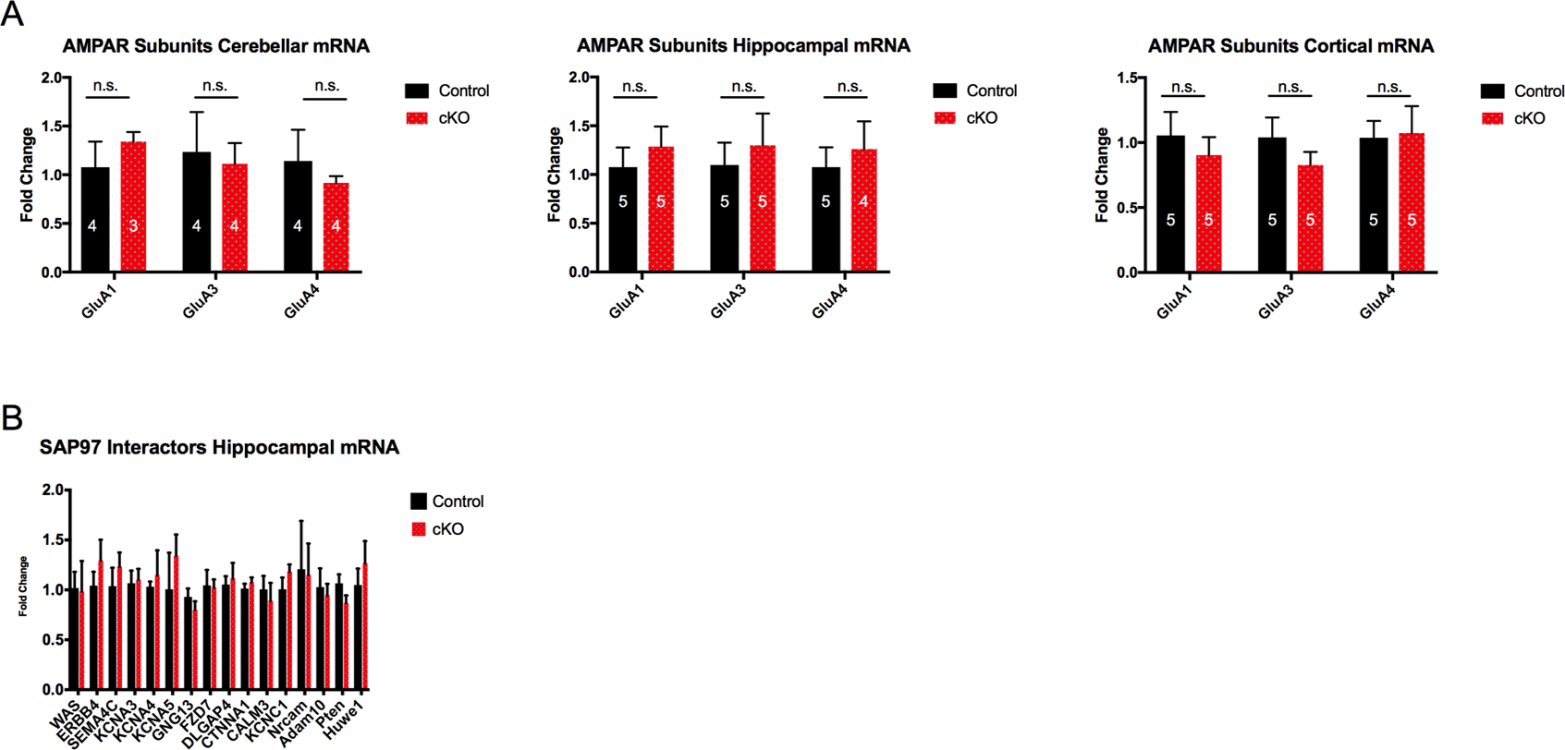
No change in mRNA expression level of AMPAR subunits and selected SAP97 interactor genes in SAP97-cKO animals. (A) qPCR results showing no significant change in abundance of *GluA1*, *GluA3*, or *GluA4* mRNA transcripts in selected brain regions. (B) qPCR results showing no significant change in abundance of mRNA levels of selected *SAP97* interactor genes in hippocampus. n.s., no significance (two-tailed Student’s *t* test). Data are presented as mean ± SEM.

*SAP97* is a scaffolding protein that allows for a large number of protein-protein interactions. Thus, the absence of *SAP97* could potentially affect the expression level of numerous proteins. To determine whether loss of *SAP97* contributes to changes in expression levels of other identified members of the postsynaptic density, we conducted a directed qPCR screen. We measured mRNA levels firstly of all AMPAR subunits, as *SAP97* is known to be the only Dlg-MAGUK to directly bind *GluA1*. mRNA levels of *GluA1*, *GluA3*, and *GluA4* remained unchanged in the three brain regions that were probed (cerebellum, hippocampus, and cortex) (Fig 3A). Results from *GluA2* were highly variable and thus removed from the study.

We next sought to determine whether the levels of other proteins known to interact with *SAP97* were affected by loss of *SAP97*. We measured mRNA levels of 16 genes in the hippocampus. From our selection of 16 genes, we observed no differences in the mRNA expression level between control and SAP97-cKO animals (Fig 3B). These results would suggest that the abundance of genes from our selection is not significantly regulated by *SAP97* expression.

### Identification of SAP97-regulated transcripts in the hippocampus

Given that we observed no group differences in our directed qPCR screen, we sought a broader, unbiased approach by performing RNAseq analysis on hippocampi from SAP97-cKO and control mice (n = 4 per group). For each animal, we verified the presence or absence of *SAP97* by western blot on brain tissue before submitting hippocampal samples for sequencing.

**Fig 4:**
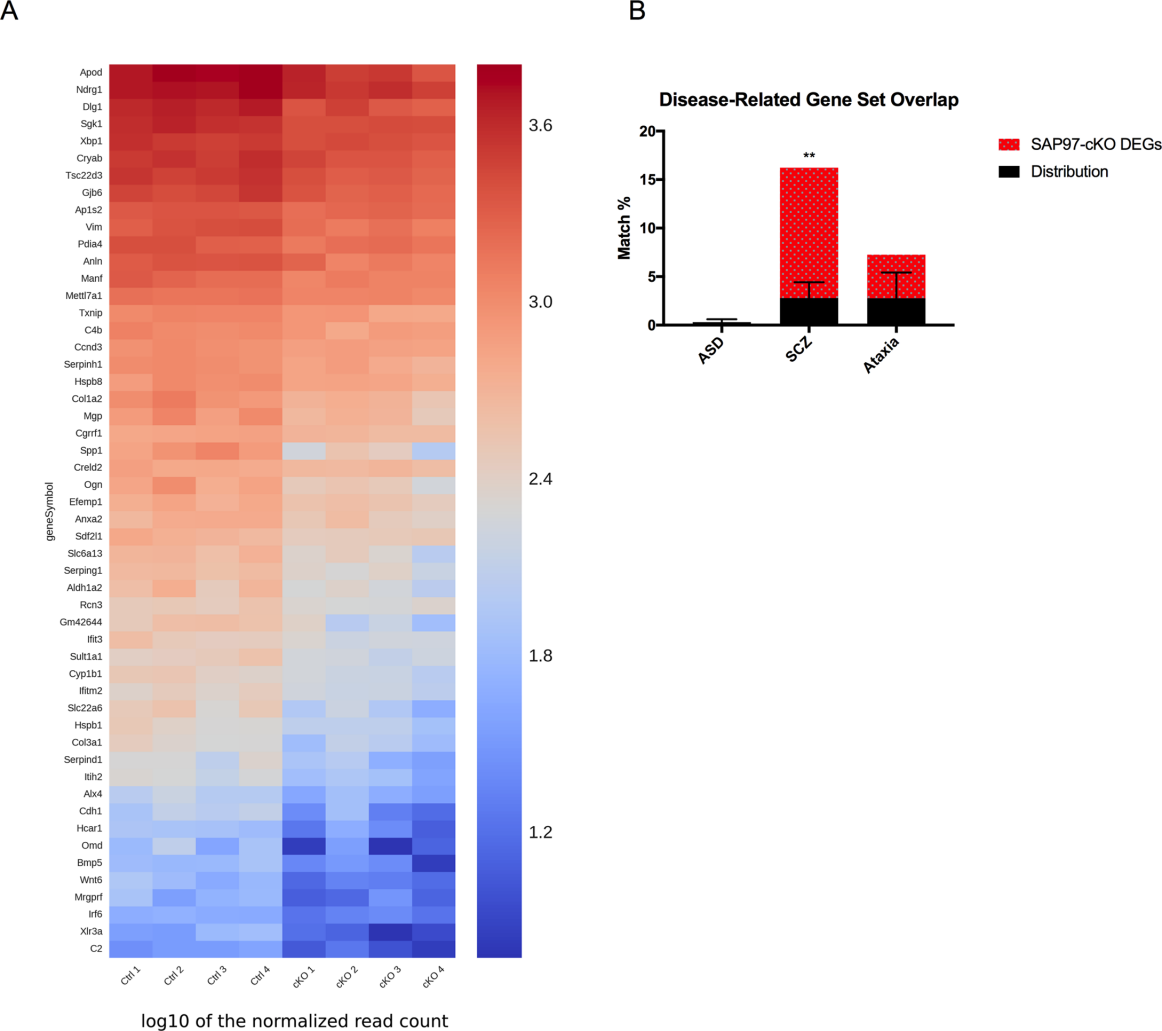
Loss of SAP97 leads to downregulation of DEGs and enrichment of SCZ risk-related genes. (A) Heat map representation of downregulation of DEGs in SAP97-cKO hippocampus. (B) DEGs are specifically enriched for SCZ risk-related genes. *P < .05 (two-tailed Student’s *t* test), **P < .01 (hypergeometric probability test).

A total of 66 genes were found to be significantly downregulated in the SAP97-cKO animals as compared to control hippocampi (FDR < 0.25) (Fig 4A, Table 1). In contrast, only one gene was upregulated in the hippocampi of SAP97-cKO animals as compared to control hippocampi. Gene ontology analysis of the DEGs revealed enrichment for numerous cellular and molecular functional categories, including those related to “Cell Morphology,” “Cellular Development,” and “Cell-To-Cell Signaling and Interaction” (Tables 2 and 3). Additionally, the top enriched ID Associated Network Functions included “Cellular Development, Cellular Growth and Proliferation, Hematological System Development and Function,” and “Developmental Disorder, Embryonic Development, Organ Development” (Table 4). Gene ontology terms to describe gene products known to be associated with ASD or the neurexin-neuroligin-SHANK complex in mice frequently include “Cell Communication” and “Nervous System Development”, which overlaps with the findings in our RNAseq study [36]. Previous studies that have conducted RNAseq on SCZ patients and performed gene ontology analysis on the resulting DEGs have identified regulation of the actin cytoskeleton as a key pathway [6]. While the actin cytoskeleton was not directly implicated by our RNAseq study, it is essential for many of the gene ontology analysis terms listed in our data set. As proper arrangement of the actin cytoskeleton is essential for neuronal cell maturation and migration, neurite outgrowth, and maintenance of synaptic density and plasticity, dysregulation of these pathways in the nervous system could have severe consequences in psychiatric disorders such as SCZ.

**Table 1:** List of genes with significant expression differences between control and SAP97-cKO mice.

Ensemble Genome ID	Gene Symbol	Log Fold Change
ENSMUSG00000019970	Sgk1	0.604982801
ENSMUSG00000022770	Dlg1	0.920172279
ENSMUSG00000023224	Serping1	1.092300119
ENSMUSG00000029304	Spp1	2.086028102
ENSMUSG00000031431	Tsc22d3	0.662945543
ENSMUSG00000021390	Ogn	-1.36634343
ENSMUSG00000026728	Vim	-0.73795218
ENSMUSG00000022769	Sdf2l1	0.813450163
ENSMUSG00000020467	Efemp1	0.785266079
ENSMUSG00000033227	Wnt6	1.860240362
ENSMUSG00000055128	Cgrrf1	0.544558433
ENSMUSG00000037254	Itih2	-1.44816667
ENSMUSG00000070691	Runx3	2.433336132
ENSMUSG00000032575	Manf	0.581728345
ENSMUSG00000031289	Il13ra2	1.998579379
ENSMUSG00000029661	Col1a2	1.062558267
ENSMUSG00000067038	Rps12-ps3	2.467941625
ENSMUSG00000054619	Mettl7a1	-0.39870621
ENSMUSG00000024650	Slc22a6	1.704915114
ENSMUSG00000015090	Ptgds	1.253057418
ENSMUSG00000026043	Col3a1	1.300397001
ENSMUSG00000030357	Fkbp4	0.343944084
ENSMUSG00000071005	Ccl19	2.363646432
ENSMUSG00000105843	Gm42644	-1.52239332
ENSMUSG00000030711	Sult1a1	0.875568486
ENSMUSG00000079293	Clec7a	2.007730582
ENSMUSG00000070436	Serpinh1	0.658564574
ENSMUSG00000030154	Klrb1f	2.174662835
ENSMUSG00000004951	Hspb1	1.133328217
ENSMUSG00000036777	Anln	0.750523147
ENSMUSG00000027248	Pdia3	0.350126593
ENSMUSG00000030218	Mgp	1.053034845
ENSMUSG00000030108	Slc6a13	1.258734628
ENSMUSG00000057836	Xlr3a	2.154616401
ENSMUSG00000000303	Cdh1	1.987938463
ENSMUSG00000024087	Cyp1b1	-0.95956574
ENSMUSG00000032231	Anxa2	0.831693618
ENSMUSG00000060591	Ifitm2	-0.82147731
ENSMUSG00000049241	Hcar1	1.730353275
ENSMUSG00000032060	Cryab	0.561687665
ENSMUSG00000026638	Irf6	1.238846117
ENSMUSG00000022548	Apod	0.868596841
ENSMUSG00000013584	Aldh1a2	1.291353747
ENSMUSG00000019539	Rcn3	0.615255839
ENSMUSG00000040055	Gjb6	-0.57564983
ENSMUSG00000040310	Alx4	1.225356858
ENSMUSG00000031367	Ap1s2	0.377838987
ENSMUSG00000023272	Creld2	-0.51269519
ENSMUSG00000038155	Gstp2	-1.96171475
ENSMUSG00000107215	Gm43197	2.106413397
ENSMUSG00000074896	Ifit3	0.830338737
ENSMUSG00000020484	Xbp1	0.420933711
ENSMUSG00000034435	Tmem30b	2.348757156
ENSMUSG00000022766	Serpind1	1.521272379
ENSMUSG00000005125	Ndrg1	0.558146377
ENSMUSG00000038393	Txnip	0.584362558
ENSMUSG00000041548	Hspb8	0.559621993
ENSMUSG00000034165	Ccnd3	0.399017781
ENSMUSG00000027048	Abcb11	-2.19248413
ENSMUSG00000073418	C4b	0.516645555
ENSMUSG00000025823	Pdia4	-0.55418986
ENSMUSG00000031070	Mrgprf	1.766073987
ENSMUSG00000032179	Bmp5	1.738362368
ENSMUSG00000043795	Prr33	2.024615333
ENSMUSG00000048368	Omd	2.520720092
ENSMUSG00000066861	Oas1g	2.341011247
ENSMUSG00000024371	C2	1.517189655

**Table 2:** List of top diseases identified through IPA that were affected in hippocampus of SAP97-cKO animals.

Name	p-value	Genes Affected
Organismal Injury and Abnormalities	2.58E-02–1.30E-05	37
Respiratory Disease	2.52E-02–1.30E-05	10
Endocrine System Disorders	2.52E-02–1.42E-05	16
Gastrointestinal Disease	2.21E-02–1.42E-05	21
Immunological Disease	2.52E-02–1.42E-05	14

**Table 3:** List of top molecular and cellular functions identified through IPA that were affected in hippocampus of SAP97-cKO animals.

Name	p-value	Genes Affected
Cell Death and Survival	2.83E-02–5.79E-05	21
Cellular Movement	2.52E-02–6.58E-05	18
Cell Morphology	2.52E-02–2.70E-04	19
Cellular Development	2.52E-02–2.70E-04	25
Cell-To-Cell Signaling and Interaction	2.47E-02–3.32E-04	17

**Table 4:** List of top networks identified through IPA that were affected in hippocampus of SAP97-cKO animals.

ID Associated Network Functions	Score
Organismal Injury and Abnormalities, Respiratory Disease, Cellular Movement	35
Cellular Development, Cellular Growth and Proliferation, Hematological System Development and Function	22
Ophthalmic Disease, Organismal Injury and Abnormalities, Hereditary Disorder	22
Cell Cycle, Gene Expression, Skeletal and Muscular System Development and Function	5
Developmental Disorder, Embryonic Development, Organ Development	2

### Schizophrenia risk enrichment in DEG set

Given that *SAP97*, along with the other members of the Dlg-MAGUK family, have been strongly implicated in psychiatric disorders such as ASD and SCZ, we chose to examine whether the DEG set was enriched for disease risk-associated genes. In order to determine whether the DEG set had a significant overlap with genes implicated in psychiatric disorders such as ASD and SCZ, we compared our DEG set with disease-related gene databases. For determining overlap with ASD-related genes, we used the gene set previously generated from the transmission and *de novo* association test (TADA), which consists of 107 genes. When we matched our DEG list to the TADA ASD gene list, we did not find the match percentage to be significant based on the hypergeometric distribution (Distribution mean = 0.30, standard deviation = 0.30; SAP97-cKO DEG 0.0) (Fig 4B). We next chose to compare our DEG list to SCZ risk-related genes found from SZDB: A Database for Schizophrenia Genetic Research (szdb.org). The distilled list of genes from this database gives a score for each gene based on criteria such as convergent functional genomics, copy number variation, differential expression, genome wide association study, and linkage and association studies. The more categories a certain gene is implicated in, the higher the score for that gene. Based on this model, we chose the top 1,000 genes from this database to match to our SAP97-cKO DEG list. Interestingly, we found the SAP97-cKO DEG list to have a significant amount of overlap to the SZDB list based on the hypergeometric test (Distribution mean = 2.79, standard deviation = 1.63; SAP97-cKO DEG 13.43, p = 0.0018) (Fig 4B). Finally, we matched our SAP97-cKO DEG list to ataxia risk-related genes as a negative control, as ataxia is not classified as a neuropsychiatric disorder and *SAP97* has not previously been implicated in ataxia. We used a list of ataxia risk-related genes compiled from GeneDx, whose clinical team compiled using multiple sources, including Online Mendelian Inheritance in Man (OMIM), Human Gene Mutation Database (HGMD), and Human Phenotype Ontology (HPO) terms. The total number of genes in this list was 993, which would also allow us to control for the size of the SCZ gene list used. When we compared our SAP97-cKO DEG list to the GeneDx ataxia gene set, we found no significant match percentage (Distribution mean = 2.77, standard deviation = 2.65; SAP97-cKO DEG 4.48) (Fig 4B). Together, these results suggest that SAP97-cKO DEGs are specifically enriched for SCZ risk-related genes.

### Behavioral analysis of SAP97-cKO mice

Next, we performed a battery of behavioral tests to screen for behavioral deficits in the SAP97-cKO mice.

#### Mild exploratory behavior deficit in female SAP97-cKO animals

**Fig 5:**
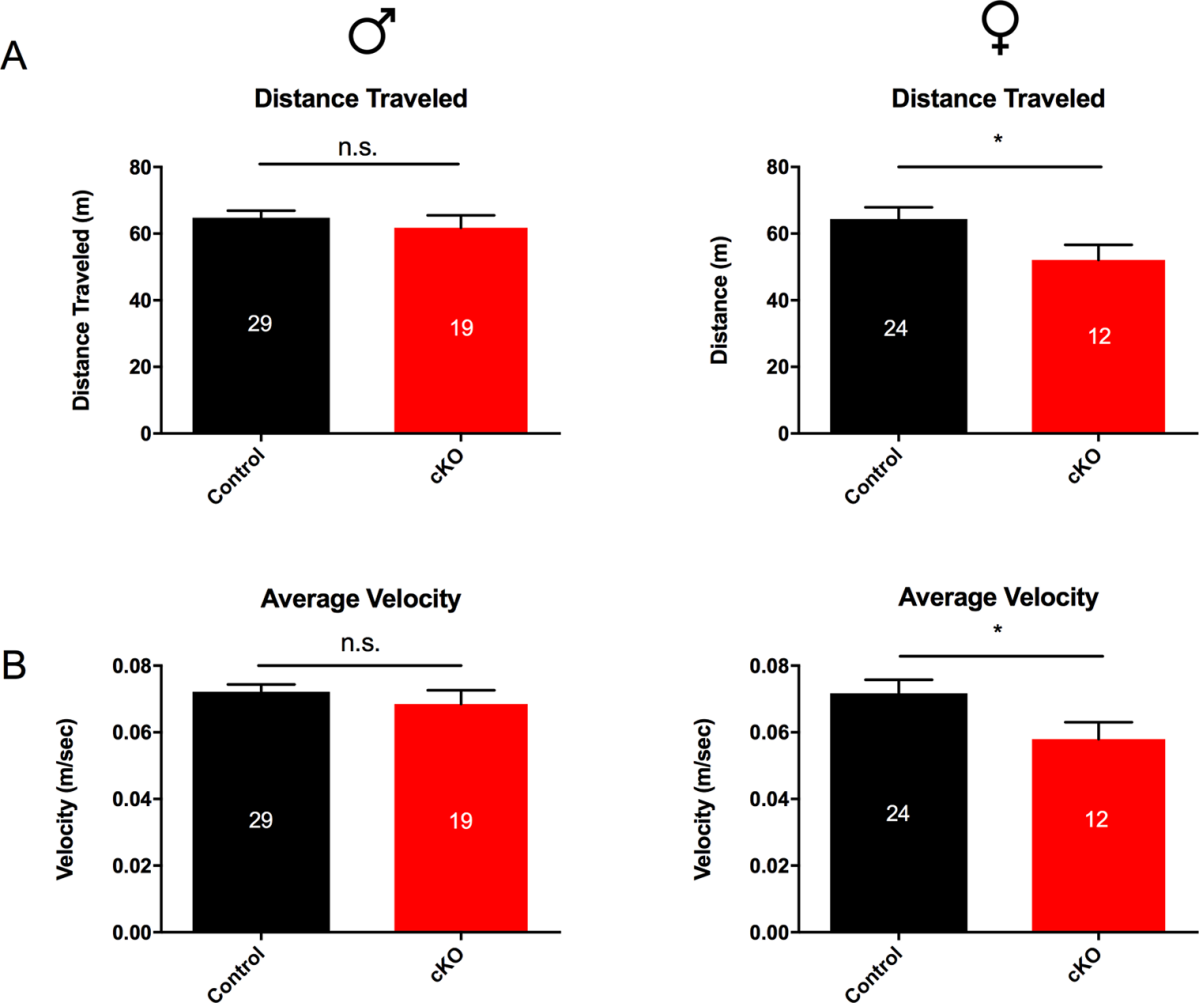
Open field behavior indicates female-specific deficit in exploratory motor behavior of SAP97-cKO animals. (A) No group differences seen in average distance traveled in male animals. Female SAP97-cKO animals display significantly less distance traveled. (B) No group differences seen in average speed in male animals, while female SAP97-cKO show decreased speed. n.s., no significance, *P < .05, **P < .01 (two-tailed Student’s *t* test). Data are presented as mean ± SEM.

We first performed the open field test to examine general ambulation. In the males, we observed no change in the total distance traveled (Ctrl 64.8 ± 2.102, n = 29; SAP97-cKO 61.76 ± 3.763, n = 19) (Fig 5A) or the speed of the animals (Ctrl 0.07214 ± 0.002259, n = 29; SAP97-cKO 0.06847 ± 0.004183, n = 19) (Fig 5B). This indicates that the male SAP97-cKO mice do not have a basic impairment in movement. However, female SAP97-cKO animals exhibited decreases in total distance traveled (Ctrl 64.39 ± 3.495, n = 24; SAP97-cKO 52.1 ± 4.519, n = 12, p = 0.0446) (Fig 5A) and speed (Ctrl 0.0717 ± 0.004071, n = 24; SAP97-cKO 0.058 ± 0.005053, n = 12, p = 0.0497) (Fig 5B). These observations may indicate either a motor deficit or an impairment in exploratory behavior that is specific to female SAP97-cKO animals.

#### No changes in anxiety levels in SAP97-cKO animals

**Fig 6:**
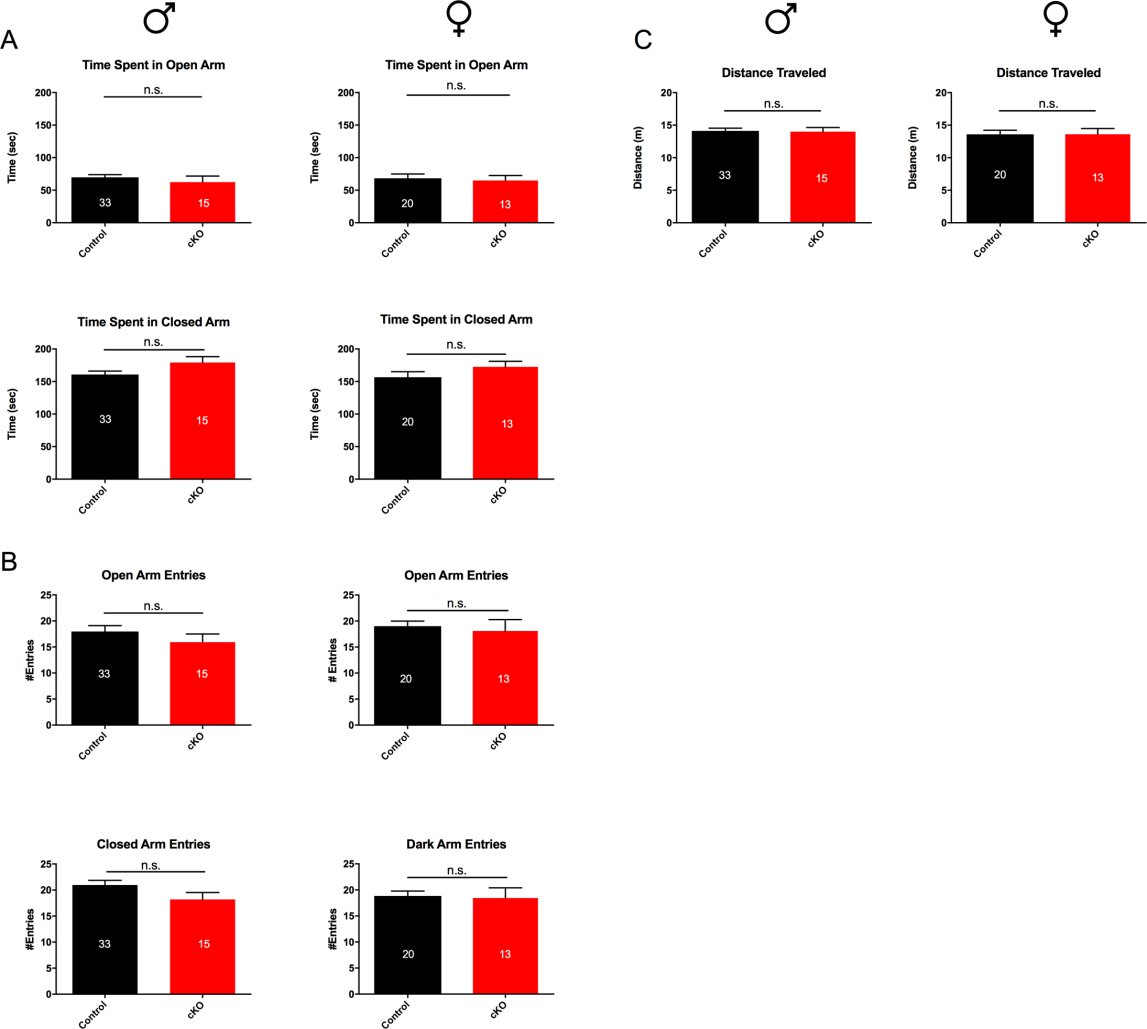
Comparison of elevated plus maze behavior between control and SAP97-cKO animals. (A) No group differences seen in average total time spent in open arms vs closed arms of maze. (B) No group differences seen in total open arm entries or closed arm entries. (C) No group differences seen in average distance traveled in elevated plus maze apparatus. n.s., no significance (two-tailed Student’s *t* test). Data are presented as mean ± SEM.

We next chose to examine anxiety-like behavior. In order to gauge whether SAP97-cKO mice had alterations in anxiety-like behavior, we performed the standard elevated plus maze. When comparing time spent in open arms versus the closed arms, we saw no significant differences between genotypes in both males (open arms: Ctrl 69.75 ± 4.098, n = 33; SAP97-cKO 62.47 ± 9.331, n = 15; closed arms: Ctrl 160.8 ± 5.339, n = 33; SAP97-cKO 179.3 ± 8.893, n = 15) and females (open arms: Ctrl 68.17 ± 6.734, n = 20; SAP97-cKO 64.85 ± 7.72, n = 13; closed arms: Ctrl 156.6 ± 8.515, n = 20; SAP97-cKO 172.5 ± 8.559, n = 13) (Fig 6A). Likewise, the number of entries into the open versus closed arms was similar between genotypes of both sexes (Fig 6B). Total distance traveled in the maze was also measured and compared between Ctrl and SAP97-cKO animals to ensure no significant differences in overall exploration of the maze (Fig 6C). These observations indicate no obvious anxiety-like phenotype in the SAP97-cKO animals.

#### Mild male-specific cognitive deficit in SAP97-cKO mice

**Fig 7:**
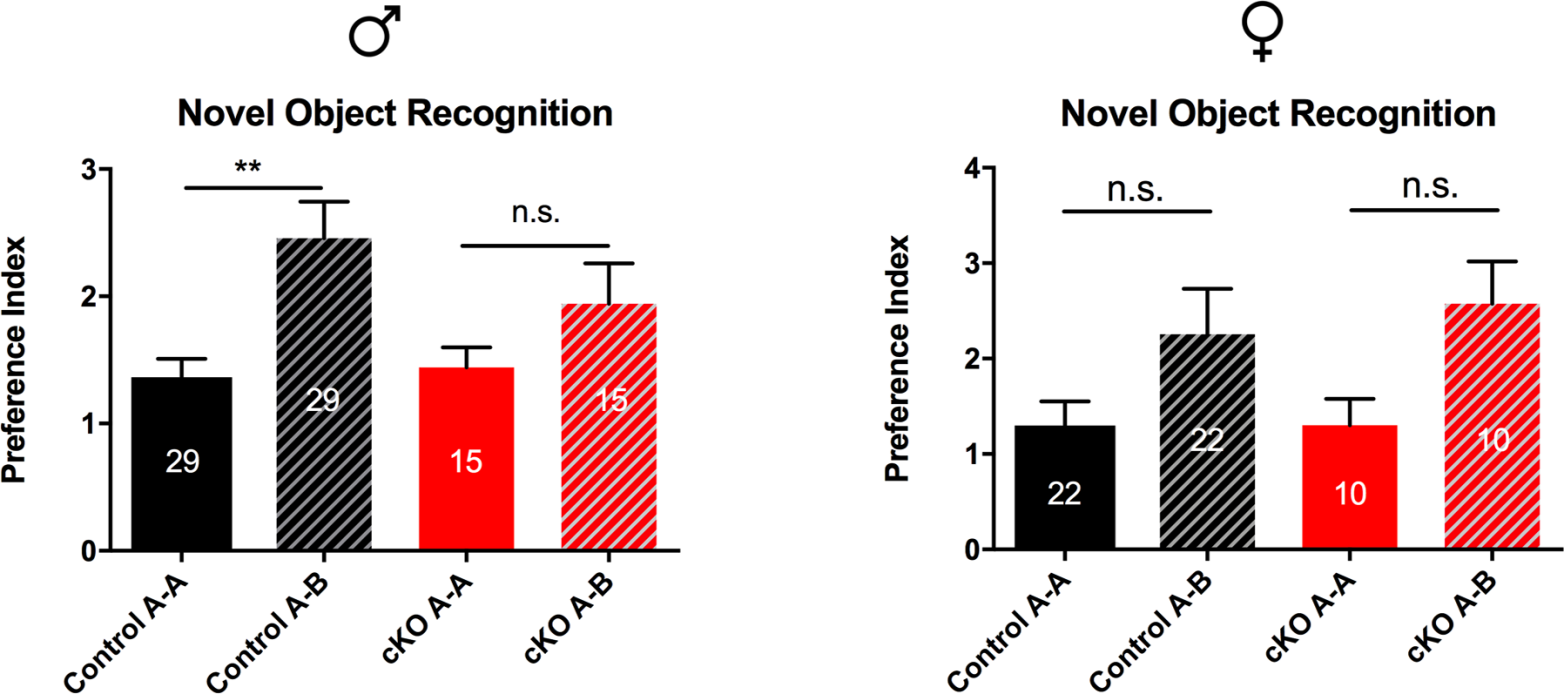
Comparison of novel object recognition behavior indicates male-specific cognitive deficit. Control male animals exhibit preference for novel object (Ctrl A-A vs Ctrl A-B), while SAP97-cKO male animals do not show preference. Both control and SAP97-cKO female animals show trend for preference of novel object, but did not reach significance. n.s., no significance, **P < .01 (ordinary one-way ANOVA with Tukey’s test for multiple comparisons). Data are presented as mean ± SEM.

We next chose to examine cognitive behavior in the SAP97-cKO mice. The novel object recognition task is a standard test for cognition that measures ability to recall an object previously observed, as indicated by preference for a novel object (see Methods). During the training phase of this task, we observed no significant differences between the ratio of time spent investigating the two identical objects A and A’ for both males and females (Male: Ctrl 1.364 ± 0.1438, n = 29; SAP97-cKO 1.441 ± 0.1578, n = 15; Female: Ctrl 1.3 ± 0.2506, n = 22; SAP97-cKO 1.301 ± 0.2775, n = 10) indicating that the animals had no prior bias. During the testing phase, control male mice displayed a marked increase in the preference index for the novel object, while SAP97-cKO male mice showed no significant increase in novel object preference index (Ctrl A-A 1.364, Ctrl A-B 2.457; SAP97-cKO A-A 1.441, SAP97-cKO A-B 1.941, *F*_*(3*, *79)*_ = 5.311, p = 0.0022) (Fig 7). When we examined this behavior in the females, we observed a trending, but not significant, increase in preference index for the novel object in both control and SAP97-cKO animals (Ctrl A-A 1.3, Ctrl A-B 2.255; SAP97-cKO A-A 1.301, SAP97-cKO A-B 2.575, *F*_*(3*, *57)*_ = 2.59, p = 0.0616) (Fig 7). These findings suggest a modest male-specific cognitive deficit in the SAP97-cKO animals.

#### Female-specific motor learning deficit in SAP97-cKO mice

**Fig 8:**
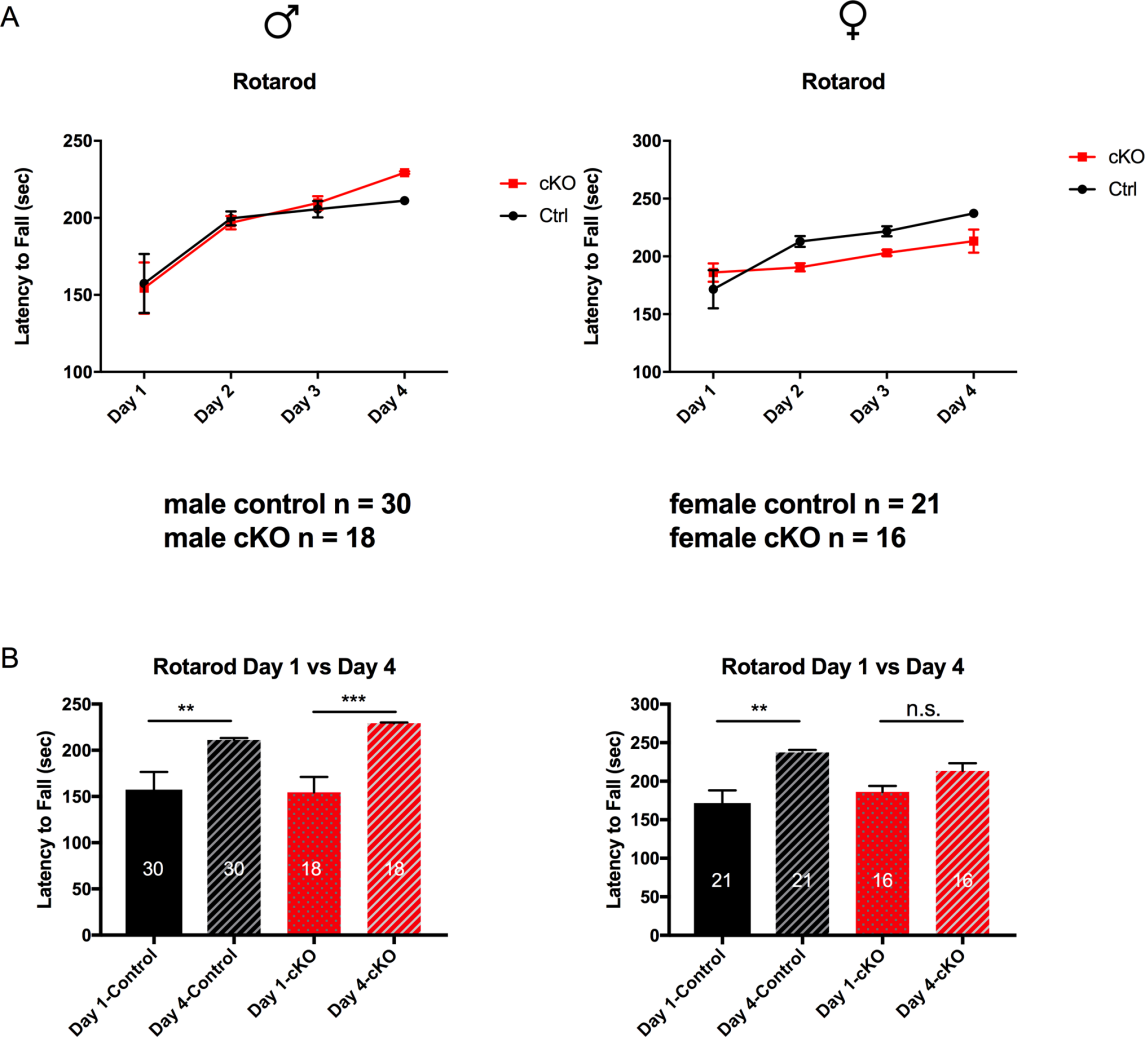
Comparison of rotarod behavior indicates female-specific motor learning deficit. (A) Both control and SAP97-cKO male animals show learning over the 4-day course of rotarod paradigm. Control female animals show increased motor learning over course of 4 days, while female SAP97-cKO show no significant increase in motor learning. (B) Plots showing comparison of Day 1 versus Day 4 rotarod data for control and SAP97-cKO animals indicates female-specific motor learning deficit. n.s., no significance, **P < .01, ***P < .001 (repeated-measures two-way ANOVA with Tukey’s test for multiple comparisons). Data are presented as mean ± SEM.

We next chose to examine motor learning behavior in the SAP97-cKO mice. In order to determine whether this behavioral change is present in the SAP97-cKO mice, we performed the standard rotarod task (see Methods). Analysis of both sexes showed significant time effects (Male: *F*_*(3*, *6)*_ = 12.31, p = 0.0057; Female: *F*_*(3*, *6)*_ = 9.126, p = 0.0118), while only female animals showed a trend for genotype effects and significant time and genotype interaction effects (genotype effect: *F*_*(1*, *2)*_ = 11.53, p = 0.0769; time x genotype effect: *F*_*(3*, *6)*_ = 5.099, p = 0.0434) (Fig 8A). Control animals of both sexes showed a significant increase in latency to fall from the rod from day 1 to day 4 (Male: Day 1 157.5 ± 19.12, Day 4 211.1 ± 2.074, n = 30, p = 0.0010; Female: Day 1 171.6 ± 16.53, Day 4 237.3 ± 3.439, n = 21, p = 0.0021), indicating learning of the task (Fig 8A and 8B). However, while male SAP97-cKO mice showed no learning impairment (Day 1 154.4 ± 16.64, Day 4 229.4 ± 0.8372, n = 18, p = 0.0002), female SAP97-cKO mice showed no significant learning over the timecourse of this task (Day 1 186 ± 7.927, Day 4 213.3 ± 9.988, n = 16) (Fig 8A and 8B). These results suggest that there is a female-specific motor learning or coordination deficit present in the SAP97-cKO mice.

#### No social deficits present in SAP97-cKO mice

**Fig 9:**
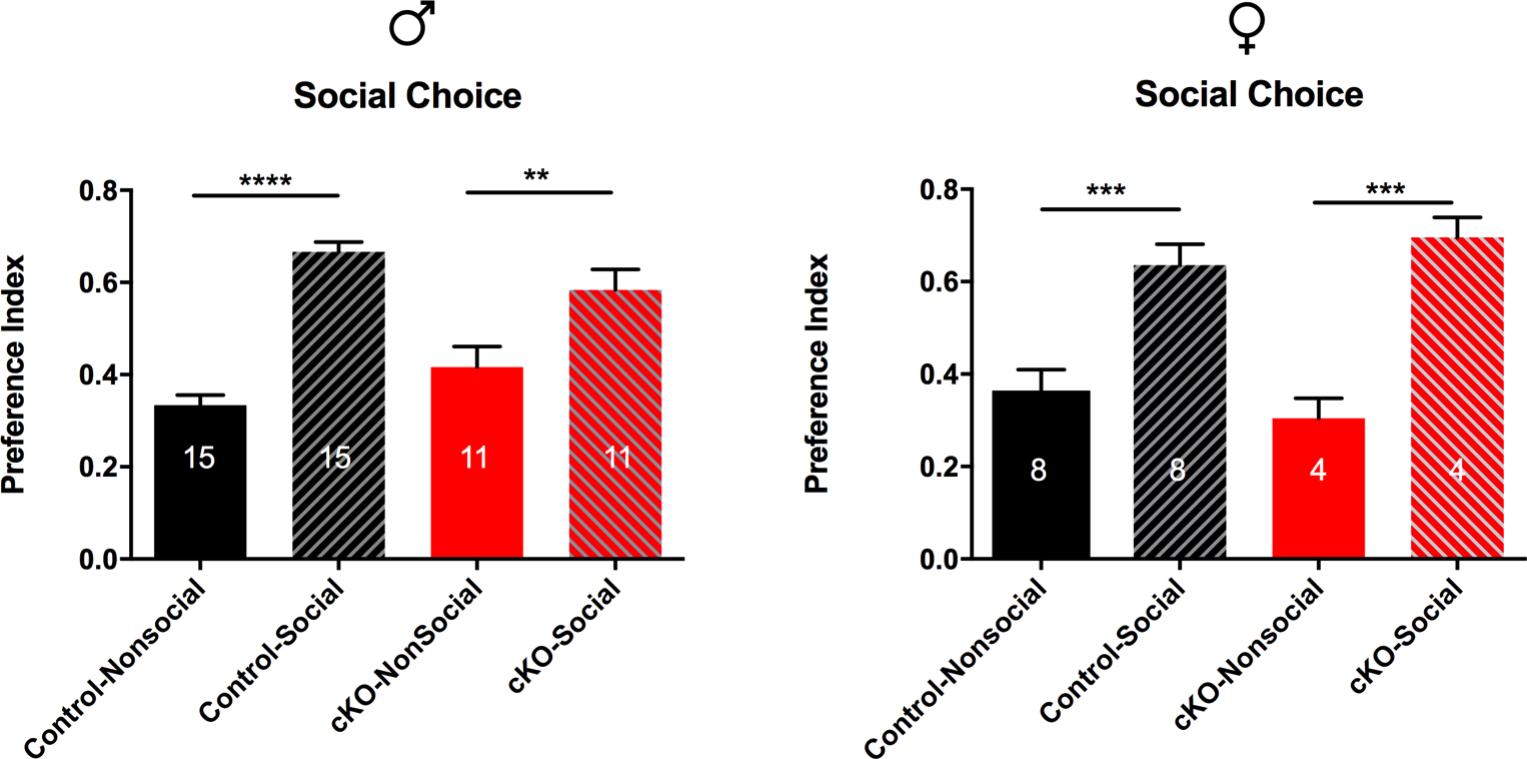
Comparison of social choice behavior between control and SAP97-cKO animals. No significant differences observed between control and SAP97-cKO male or female animals in preference for social target. **P < .01, ***P < .001, ****P < .0001 (ordinary one-way ANOVA with Tukey’s test for multiple comparisons). Data are presented as mean ± SEM.

The last behavior we chose to examine in the SAP97-cKO mice was sociability. We looked for changes in this behavioral domain by using the three-chambered social choice paradigm (see Methods). During the testing phase of this paradigm, we measured preference for the social target zone versus the nonsocial target zone. Control and SAP97-cKO animals of both sexes exhibited a strong preference for spending time in the social target zone (Male: Ctrl-Nonsocial 0.334, Ctrl-Social 0.666, n = 15; SAP97-cKO-Nonsocial 0.4164, SAP97-cKO-Social 0.5836, n = 11, *F*_*(3*, *48)*_ = 24.67, p<0.0001; Female: Ctrl-Nonsocial 0.3641, Ctrl-Social 0.6359, n = 8; SAP97-cKO-Nonsocial 0.3045, SAP97-cKO-Social 0.6955, n = 8, *F*_*(3*, *20)*_ = 14.53, p<0.0001) (Fig 9). These results suggest no social deficit in the SAP97-cKO mice.

## Discussion

*SAP97* is a member of the Dlg-MAGUK family, a family of genes that plays an important role in synapse biology and has repeatedly been implicated in neuropsychiatric disorders [7,21,23,37]. In this study, we aim to address the direct role of *SAP97* in overall behavior and its potential for contributing to pathology. We generated and studied mice that were null for *SAP97* in the nervous system and make three principal observations. First, there are no compensatory changes in expression levels of other Dlg-MAGUKS or AMPARs in the SAP97-cKO versus controls. Second, loss of *SAP97* is associated with moderate changes in gene transcripts related to SCZ. And third, SAP97-cKO animals display a mild behavioral phenotype, consisting of a male-specific cognitive deficit and female-specific motor deficit. While future behavioral experiments will be necessary to understand whether *SAP97* regulates SCZ-specific behaviors, our results indicate that *SAP97* probably plays a limited role in organismal behavior under standard laboratory conditions and its absence leads to transcriptomic changes associated with SCZ.

### ASD and SCZ as polygenic disorders

Investigations of ASD, SCZ, and other related psychiatric disorders indicate a highly polygenic architecture with small effect sizes of each implicated risk variant. Mouse modeling of these disorders by targeting one such risk variant typically demonstrates a moderate, or incomplete manifestation of the human disorder. This is well illustrated by human and mouse studies of the *ProSAP/SHANK* family member *SHANK3*. Human genetic studies link mutations in *SHANK3* to a broad range of neuropsychiatric disorders. For example, deletions of exons 1–9 or exons 1–17 of *SHANK3* have been found in patients exhibiting severe language delay and significant intellectual disability. Mice generated to mimic these deletions were generated by Peca et al. and the main behavioral effects were repetitive grooming and deficits in social interaction [9]. Jiang et al. used a different targeting strategy to mimic the human deletions and the mice displayed repetitive behaviors, deficits in social interaction, abnormal ultrasonic communication patterns and learning and memory deficits [38]. In a second well-studied family, affected individuals displayed ASD-features and this was linked to a deletion of *SHANK3* exon 21 (an exon that included the Homer binding domain). Mice generated to mimic this genetic lesion were created by Kouser and Speed et al. and ~2.5 month old animals exhibit defects in spatial learning and memory, motor-coordination deficits, hypersensitivity to heat, novelty avoidance, but minimal social abnormalities and no repetitive grooming behavior [11]. Together, this work demonstrates that creating a mouse with a genetic lesion that closely mimics, or is identical, to the gene defect in humans with neuropsychiatric disease only partially recapitulates the human behavioral phenotypes.

This disparity between genetic lesions associated with psychiatric phenotypes and mice created to mimic the human condition also extends to the Dlg-MAGUK family. Nonsynonymous missense mutations in the Dlg-MAGUK family members have been found in ASD and SCZ patients, and decreased protein expression of *PSD-95*, *PSD-93*, and *SAP97* has been observed in the cortex of postmortem SCZ patients. To model with in mice, null alleles of *PSD-95*, *PSD-93*, and *SAP102* have been created—*PSD-95* and *SAP102* knockout animals share spatial learning memory deficits [20,39], while animals null for *PSD-95* or *PSD-93* share a hyper-social phenotype [15]. *PSD-95* and *SAP102* knockout animals display a mild, and *PSD-93* knockout animals display a severe, motor function defect [15,20]. The *PSD-95* null mouse has been the most extensively investigated animal. Feyder et al. characterized the *PSD-95* knockout mice and the mice exhibit increased repetitive behaviors, abnormal communication, hyper-social behavior, impaired motor coordination, and increased stress-reactivity and anxiety-related responses [16]. The extent to which the *PSD-95* null mice faithfully report on the contribution of *PSD-95* to psychiatric disease is an open question. Our behavioral characterization of the SAP97-cKO animals was conducted broadly as a first-pass assessment of behavioral changes. We are unable to conclude that the behavioral abnormalities observed in our study are disease specific, and further behavioral tests that are specific to ASD and SCZ will be necessary to claim whether *SAP97* directly contributes to a partial manifestation of disease phenotype. Nevertheless, our behavioral results provide a framework for understanding the role of *SAP97* in organismal behavior and brain function.

### SAP97 splice variants and their differing roles in the nervous system

*SAP97* has wide molecular diversity, which is created by extensive alternative splicing. The two most well-studied *SAP97* splice variants are *SAP97α and SAP97β*. In *SAP97α*, the prototypic N-terminal L27 domain is replaced with a putative palmitoylation motif. Overexpression of *SAP97α* (but not *SAP97β*) was shown to enhance the synaptic levels of AMPARs and to compensate for the shRNA-mediated loss of *PSD-95* in organotypic slices [40]. *SAP97* isoform-specific biology may also extend into human SCZ data. Uezato and colleagues identified a new *SAP97* splicing variant that is transcribed from a previously unreported 95-base-pair exon (exon 3b). In post-mortem prefrontal cortices of patients with SCZ, mRNA expression of exon 3b was significantly reduced, specifically in patients with early-onset SCZ [37]. A more recent genetic association analysis detected an association between SCZ and single nucleotide polymorphisms located within exon 3b [22]. How reduced levels of the *SAP97* 3b transcript may be involved in the susceptibility and pathophysiology of early-onset SCZ is unknown. While our study provides a broad characterization of the effect of *SAP97* on brain function, it will be necessary to conduct future studies aimed at addressing the individual roles of prominent splice variants.

### The role of the serpin family as a molecular module in SCZ

The RNAseq study we conducted on the hippocampi of SAP97-cKO animals indicated 67 DEGs, which were specifically enriched for SCZ-related risk genes. The specific SCZ-related risk genes we identify in our data are *SERPING1*, *RUNX3*, *CLEC7A*, *SERPINH1*, *CDH1*, *AP1S2*, *XBP1*, *SERPIND1*, and *C4B*. These observations lead us to hypothesize that *SAP97* is a component of a “molecular module” of gene products that together subserve aspects of normal behavior. Further, we hypothesize that abnormalities in the operation of this molecular module give rise to select behavioral alterations. Defects in many molecular modules in aggregate manifest as the complex psychiatric disorder we recognize as SCZ. The components of this module may interact physically, functionally, developmentally, or in terms of localization. Future work will be required to elucidate: 1) how the components of this hypothesized molecular module mechanistically interact, and 2) how this impacts brain function and behavior.

Our attention is drawn to three genes that were differentially expressed in the hippocampus of SAP97-cKO mice versus controls—serine peptidase inhibitors (serpins), as this group of genes has previously been reported in the literature to be associated with SCZ [41–46]. *SERPING1* was found to be upregulated in postmortem brain tissue from SCZ patients [44,45]. Additionally, a study of adult Swedish twins enriched for SCZ showed an association between gene expression level of *SERPING1* and thickness across the cortex, a characteristic that is potentially involved in the pathogenesis of SCZ [41]. Polymorphisms in the promoter regions of genes on 22q11, a chromosomal region that has been associated with various psychiatric illnesses including SCZ, resulted in activity differences in the gene *SERPIND1* [42]. Another well-studied member of the serpin family previously implicated in SCZ, but not directly by our RNAseq data, is neuroserpin (*SERPINI1*). *SERPINI1* is restricted to regions in the brain where synaptic changes are associated with learning and memory (cortex, hippocampus, amygdala, and olfactory bulb) [46]. *SERPINI1* has also been implicated in dendrite growth, as overexpression studies in primary neurons leads to increased dendritic arborization and altered dendritic spine shape [47]. Additionally, mice with dysregulated expression of *SERPINI1* show selective reduction of locomotor activity in novel environments, anxiety-like responses, and neophobic response to novel objects [43]. These behavioral phenotypes in the *SERPINI1* deficient mice are reminiscent of the defects we see in the SAP97-cKO animals. *SERPINI1* is also a known inhibitor of the extracellular protease tissue-type plasminogen activator (*tPA*). Conditions that affect the activity of *tPA* have consistently been described in drug-naïve cases of SCZ [48–51]. Interestingly, psychotic patients on chronic warfarin therapy for deep-vein thrombosis showed remission of psychotic symptoms, indicating that defective modulation of the coagulation pathway might contribute to the pathogenesis of SCZ [52]. *C4B*, or complement component b, is another gene directly listed from our RNAseq study that has known roles in the coagulation pathway and is an important cofactor to the serine protease family. The strongest genetic association of SCZ at a population level involves variation in the Major Histocompatibility Complex (MHC) locus, where the association of SCZ with the MHC locus arises substantially from many diverse alleles of the *C4* genes [41,53,54]. These prior observations along with our findings from the SAP97-cKO RNAseq study may highlight a potential mechanism by which *SAP97* contributes to the etiology of SCZ.

### Sex-specific differences in psychiatric disease

Psychiatric disorders are characterized by substantial sex-differences in their prevalence, symptomology, and treatment response [55]. Women are more likely than men to develop dementia, panic disorder, post-traumatic stress disorder, and major depression [56,57]. Conversely, the incidence of neurodevelopmental disorders such as ASD and SCZ is higher in males [58,59]. In our study, we conducted RNAseq on male hippocampal tissue from SAP97-cKO tissue and found the resulting DEGs to be specifically enriched for SCZ risk-related gene sets. However, our behavioral screen was undertaken on both male and female SAP97-cKO animals and identified interesting sex-specific differences. This raises the possibility that the RNAseq profile of female SAP97-cKO mice may be at least partially distinct from the male SAP97-cKO dataset.

One potential limitation of our study of female behavior is the lack of assessment of the estrous cycle. Female mice in distinct stages of the estrous cycle have been previously shown to perform differently in behavioral tasks related to anxiety and cognition. Furthermore, it is thought that oestrogens play a protective role against SCZ [60]. It will be vital to perform behavioral testing at different stages of the female estrous cycle, as well as corroborate behavioral findings with RNAseq data in order to have a complete understanding of the role of *SAP97* in the female brain. Additionally, results from these future experiments may address the behavioral sex differences we observed in the SAP97-cKO animals.

### Conclusion

Our study provides the first broad behavioral and transcriptomic characterization of *SAP97* in the mouse nervous system. Despite study limitations, we show that loss of *SAP97* contributes to moderate behavioral abnormalities in both male and female animals, as well as an enrichment of SCZ related genes in the male animals. Our findings are a first step to understanding the direct role of *SAP97* in overall organismal behavior, and potentially provide a stepping-stone for understanding the molecular mechanism by which *SAP97* contributes to neuropsychiatric disorders.
